# Antitumor, Immunomodulatory and Antiangiogenic Efficacy of Medicinal Mushroom Extract Mixtures in Advanced Colorectal Cancer Animal Model

**DOI:** 10.3390/molecules25215005

**Published:** 2020-10-28

**Authors:** Boris Jakopovic, Nada Oršolić, Sandra Kraljević Pavelić

**Affiliations:** 1 Dr Myko San—Health from Mushrooms Co., Miramarska cesta 109, HR-10000 Zagreb, Croatia; borisjakopovic@yahoo.com; 2 Divison of Animal Physiology, Faculty of Science, University of Zagreb, Rooseveltov trg 6, HR-10000 Zagreb, Croatia; 3 Faculty of Health Studies, University of Rijeka, Ulica Viktora cara Emina 5, HR-51000 Rijeka, Croatia; sandrakp@uniri.hr

**Keywords:** colorectal cancer, medicinal mushrooms, immunotherapy, multi-target, combination therapy, 5-fluorouracil, survival, angiogenesis, macrophage polarization

## Abstract

Due to frequent drug resistance and/or unwanted side-effects during conventional and targeted cancer treatments, development of multi-target therapies is an important research field. Medicinal mushrooms’ isolated specific compounds and mushroom extracts have been already proven as non-toxic multi-target inhibitors of specific oncogenic pathways, as well as potent immunomodulators. However, research on antitumor effects of multiple-species extract mixtures was limited so far. The aim of this study was therefore, a study of medicinal mushroom preparations AGARIKON.1 and AGARIKON PLUS on colorectal cell lines in vitro and colorectal mice model in vivo. We found a significant antiproliferative and pro-apoptotic effect of tested medicinal mushroom preparations on colorectal (HCT-116, SW620) tumor cell lines, while the effect on human fibroblast cell line (WI-38) was proliferative emphasizing a specificity towards tumor cell lines. We further investigated the effect of the medicinal mushroom preparations AGARIKON.1 and AGARIKON PLUS in various combinations with conventional cytostatic drug 5-fluorouracil in the advanced metastatic colorectal cancer mouse model CT26.WT. AGARIKON.1 and AGARIKON PLUS exhibited immunostimulatory and antiangiogenic properties in vivo which resulted in significantly increased survival and reduction in tumor volume. The antitumor effects of AGARIKON.1 and AGARIKON PLUS, with or without 5-fluorouracil, are based on M1 macrophage polarization enhancement, inhibition of M2 and tumor-associated macrophage (TAM) polarization, effects on T helper cell Th1/Th2/Th17 cytokine profiles, direct inhibition of CT26.WT tumor growth, inhibition of vascular endothelial growth factors (VEGF) and metalloproteinases 2 and 9 (MMP-2 and MMP-9) modulation. The administration of AGARIKON.1 and AGARIKON PLUS did not show genotoxic effect. This data provides good basis for an expanded translational study.

## 1. Introduction

According to the Globocan database, 1.09 million new cases of colon and rectal cancer were diagnosed in 2018, and 551,000 died from the disease [1]. This type of tumor is thus generally third in frequency but second in mortality. The highest incidence of colon cancer has been recorded in parts of Europe (e.g., Hungary, Slovenia, Slovakia, the Netherlands and Norway), Australia and New Zealand, and in the Far East (Japan, Korea, Singapore). The incidence of colorectal cancer in various regions of the world varies six to eight times, and is significantly higher in countries with higher socioeconomic development [2,3]. Economic development in the last decade has been found to increase incidence and mortality in the Baltic countries, Russia, China and Brazil, increase in incidence but decrease in mortality in Canada, the United Kingdom, Denmark and Singapore, and decrease in incidence and mortality in the United States, Japan and France [4]. The reduction in mortality in developed countries is due to more advanced treatments as well as the introduction of prevention programs from the 1990s which result in earlier disease detection [5].

The causes of colorectal cancer (CRC) are multifactorial, and include genetic and environmental factors. Most cases of colorectal cancer are sporadic, while the hereditary component is estimated at 18–35% [6]. Among the environmental factors that can contribute to the development of the disease, the most important are high-calorie diet rich in animal fats, increased alcohol consumption, smoking as well as reduced intake of fruits, vegetables and fibers. Other risk factors include old age, male gender, insufficient physical activity as well as obesity, and inflammatory bowel diseases such as ulcerative colitis and Crohn’s disease [7]. Additionally, it is known that patients who have undergone surgery for colorectal cancer have a three times higher risk of developing new, i.e., metachronous colon cancer. The presence of adenomas is also a recognized risk factor for colorectal cancer, and tubular adenomas pose low, tubulovillous medium, and villous adenomas a high risk for CRC. Genetic syndromes, such as genetic syndromes of familial adenomatous polyposis (FAP) and Lynch syndrome (hereditary nonpolyposis colorectal cancer, HNPCC) also cause an increased CRC incidence [8]. Despite advancements in screening, approximately 35% of colorectal cancer patients present with stage IV metastatic disease at the time of diagnosis, and 20–50% with stage II or III will progress to stage IV at some point during the course of their disease [9]. Despite advancements in treatment, five-year survival rate in patients with distant metastases is only about 10–15%. Colorectal cancer is a prototypical example of a multi-stage cancer development and progression by accumulation of various mutations and epigenetic changes [10]. Current treatment of CRC includes surgery, radiation, chemotherapy and targeted therapy.

Medicinal mushroom research has identified about 800 mushroom species with significant pharmacological properties [11,12]. While more than 130 therapeutic effects of various mushroom species have been discovered, most of the research is focused on immunomodulatory and antitumor properties of mushrooms [13,14,15,16]. Antitumor immunological responses are primarily due to high-molecular weight compounds such as polysaccharides and polysaccharopeptide complexes, out of which various β-D-glucans are most well-known [17]. Direct antitumor effects are the result of dysregulation of important pathways, such as nuclear factor-kappa B (NF-κB), mitogen activated protein kinase pathway (MAPK), Akt, Wnt, Notch, p53, TGFβ, MMPs, VEGF and others, involved in tumor development and progression [18]. While most research focuses on various individual isolates from well-known mushroom species, some research indicates that combination of several mushroom species cause better anticancer effects [18,19,20]. This is the consequence of combinatorial effects of polysaccharides on the immune system, as well as of various small-molecular weight compounds (terpenoids, lactones, alkaloids, sterols and phenolic substances) on cancer-promoting pathways and processes.

Besides its known pharmacological role as a antimetabolite and pyrimidine analog, 5-fluorouracil has been shown to have immunological antitumor effects in lower doses. These effects are primarily the consequence of increase in tumor antigenicity by stimulating the production of mutational antigens [21]. Stress cell death caused by cytotoxic drugs through the activation of the DNA damage response (DDR), endoplasmic reticulum stress response and autophagy results in the release of DAMPs (danger associated molecular patterns) which in turn stimulate the activation of dendritic cells and presentation of tumor antigens to T lymphocytes [22]. Cell death associated with the formation of DAMP is called immunogenic cell death (ICD) [23]. Molecules that are ligands for NK receptors such as NKG2D and cell death receptors Fas/CD95 as well as adhesion molecules that enhance the recognition and killing of tumor cells by cytotoxic T lymphocytes are expressed on cancer cells that survive chemotherapy treatment [24]. Non-targeted immune effects of chemotherapy include the elimination of many tumor-activated immunosuppressive mechanisms mediated by immunosuppressive cytokines such as TGFβ1, inhibitory receptors such as CTLA-4, PD-1, and immune cells with immunosuppressive or tolerogenic functions such as Treg, MDSC, and M2 polarized macrophages [25]. Therefore, the rationale of using chemotherapeutics in lower, immune-stimulating doses with well-defined medicinal mushroom mixtures lays in the possible synergism between these two therapeutic approaches. This approach is of particular interest in the immunosuppressive environment of advanced tumors.

To date, there has been a limited amount of studies addressing antitumor effects of standardized medicinal mushroom blended extracts alone or in combination with standard chemotherapies. Recent meta-study of 213 studies, including 77 clinical studies that reported the use of various cytotoxic drugs, including 5-fluorouracil with *Ganoderma lucidum* or *Trametes versicolor* reported that these medicinal mushrooms enhance the efficacy and ameliorate their adverse effects, which leads to improved quality of life in cancer patients, without significant changes in pharmacokinetics of 5-fluorouracil [26]. Hence, to better understand the mechanisms of antitumor action of Agarikon.1 and Agarikon Plus preparations, with or without 5-fluorouracil in a late colorectal cancer model, we have performed in vitro and in vivo studies presented herein.

## 2. Results

### 2.1. Polyphenol and Polysaccharide Content of the Tested Extracts

Polysaccharides and polyphenols are primary active substances present in Agarikon Plus and Agarikon.1. The highest dry matter and soluble polysaccharide content was measured in tablet preparation Agarikon.1 (Table 1). On the other hand, Agarikon Plus has a much higher total polyphenol (1457.8 mg GAE/L) and flavonoid content (750.9 mg GAE/L). In liquid preparation Agarikon Plus, a significantly higher content of total phenols was found than in Agarikon.1 tablets. This may be due to the influence of excipients used in tablet production, such as silica, inulin, talc and magnesium stearate, which may interfere with the analytical process of total polyphenols determination, so that their actual proportion in tablets may be higher [27]. Additionally, a large difference in the amount of soluble polysaccharides between tablets and liquid preparations was found, with more than twice the amount of polysaccharides in tablets compared to the liquid preparation. It is known that polyphenols form complexes with proteins, polysaccharides and alkaloids, and it is possible that the formation of complexes of polyphenols and polysaccharides leads to underestimation of their quantity in tablet preparation Agarikon.1 [28,29].

### 2.2. In Vitro Analyses

**Figure 1:**
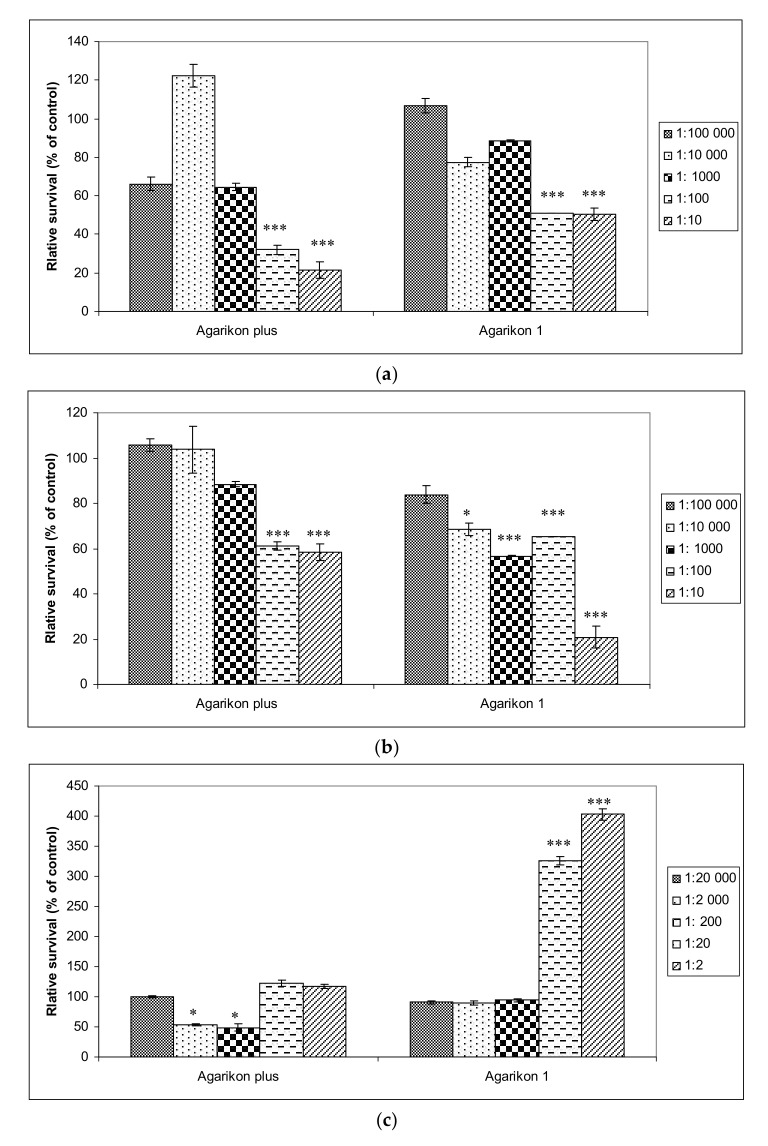
(**a**) Viability of HCT-116 and (**b**) SW620 human colorectal cancer cell lines treated with Agarikon Plus (AP) or Agarikon.1 (AG.1) (1:10–1:100,000), as well as on (**c**) human lung fibroblasts (WI-38) (1:2–1:20,000). Cell viability was determined by 3-(4,5-dimethylthiazol2-yl)-2,5-diphenyltetrazolium bromide (MTT) assay. The data are expressed as mean ± SD of cell viability in comparison with control (at 100%) from three independently performed experiments. * Significantly different (* *p* < 0.05; *** *p* < 0.001, nonparametric Kruskal–Wallis test) in comparison with control group.

Antiproliferative effects of complex extracts, Agarikon Plus and Agarikon.1 were evaluated against two tumor cell lines HCT-116 (colon adenocarcinoma), SW620 (metastatic colorectal adenocarcinoma), as well as on normal cell line WI-38 (immortalized human lung fibroblasts). In fibroblast cell line, a dilution series of 2–20,000 of Agarikon.1 and Agarikon Plus was used. Viability for HCT-116 and SW620 cell lines is shown with dilution series of 10–100,000 since the IC_50_ value was measured in this range (Figure 1). Namely, IC_50_ value for HCT-116 cell line amounts to 0.73 μg/mL for Agarikon Plus and 0.65 μg/mL for Agarikon.1. For SW620 cell line, IC_50_ is 16 μg/mL after treatment with Agarikon Plus and 0.075 μg/mL after treatment with Agarikon.1. Overall, a specific dose-dependent cytotoxic effect was observed, which was statistically significant compared to the control. Interestingly, we have established that Agarikon Plus and Agarikon.1 have proliferative effects on normal cell line WI-38, which point to a specific antiproliferative effect on tumor cell lines.

In order to examine whether specific antiproliferative effects of Agarikon.1 and Agarikon Plus on HCT-116 and SW620 tumor cell lines can be attributed to apoptosis, flow cytometry analysis was performed on these cell lines. Following the results obtained by cell viability study after treatment with Agarikon.1 and Agarikon Plus, concentrations of these extracts corresponding to dilutions of 100× (c_1_), 1000× (c_2_) and 10,000× (c_3_) relative to the stock solution were used, within which the IC_50_ concentration was determined for HCT-116 and SW620 cell lines. Since a combination of these preparations was also used in vivo, a mixture of extracts of these preparations was prepared for this experiment so that the dry matter ratio was 1:1. Cells were classified into several categories depending on the status (positive or negative) of annexin V (ANN.V) and/or propidium iodide (PI): cells in necrosis (PI+/ANN.V-), early apoptosis (PI-/ANN.V+), late apoptosis (PI+/ANN.V+).

**Figure 2:**
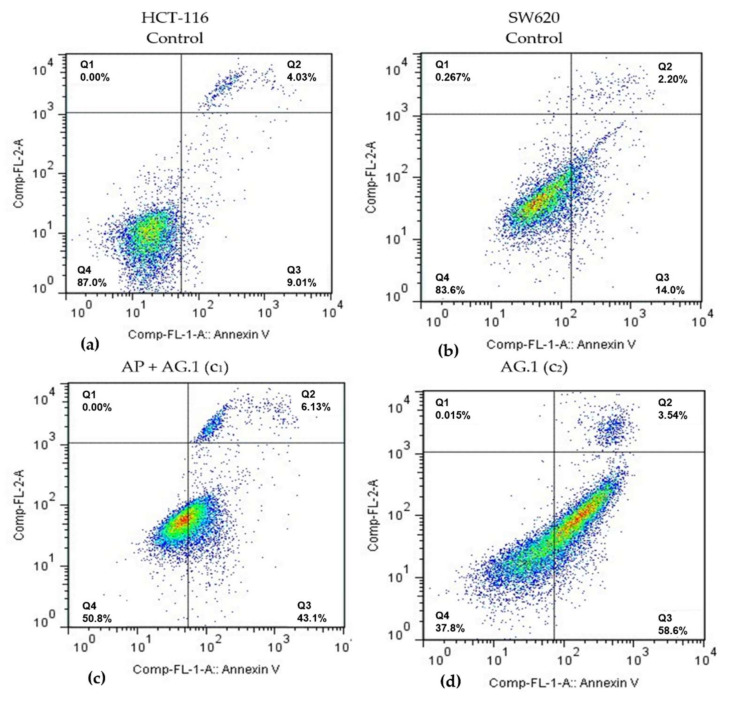
Representative diagrams of apoptotic cell diversification in HCT-116 and SW620 cells after treatment with (**c**) Agarikon Plus and Agarikon.1 (AP + AG.1) and (**d**) Agarikon.1 (AG.1) in comparison with (**a**) untreated HCT-116 and (**b**) SW620 cell lines. Quadrant legend: Q4 = lower left, An-/PI-, non-apoptotic; Q3 = lower right, An+/PI, early apoptotic; Q2 = upper right, An+/PI+, late apoptotic; Q1 = upper left, An-/PI+, necrotic.

Flow cytometry analysis of HCT-116 cell line revealed a significant apoptosis induction after 24 h treatment with medium (c_2_) and lowest (c_3_) concentrations of Agarikon.1 (AG.1) and with highest (c_1_) concentration of the combination of Agarikon.1 and Agarikon Plus (AP + AG.1) (Table 2, Figure 2). Thus, it was found that in comparison to control, where 7.2 ± 0.7% of cells are in early apoptosis, treatment with medium (c_2_) concentration of Agarikon.1 for 24 h caused this value to increase to 44.3 ± 8.5%. The proportion of cells in early apoptosis treated with the highest (c_1_) concentration of Agarikon.1 could not be determined due to precipitate formation that interfered with the assay. Cells treated with the highest concentration of Agarikon Plus (c_1_) exhibited early apoptosis in 16.24 ± 2.7% of cells, which is twice as many as the control, but not statistically significant. Statistical significance in comparison with control was also observed following treatment with a combination of Agarikon Plus and Agarikon.1 where the proportion of cells in early apoptosis was 46.56 ± 14.8%. The change in the proportion of HCT-116 cells in late apoptosis compared to control (2.46 ± 1.09%) is greatest after treatment with medium (10.1 ± 0.51%) and lowest (11.78 ± 2.55%) concentrations of Agarikon.1. The proportion of cells in early and late apoptosis treated with a combination of extracts at the highest dose was 51.06 ± 16.42% and is statistically significant. Results indicate a dose-dependent decrease in the proportion of apoptotic cells after treatment with c_2_ (26.64 ± 3.96%) and c_3_ (19.51 ± 8.36%) of Agarikon Plus with Agarikon.1. There is no statistically significant difference in the proportion of necrotic cells between control and treated cells.

Flow cytometry analysis of SW620 cells has shown that treatment with medium (c_2_) and lowest (c_3_) concentrations of Agarikon.1 (AG.1) as well as with highest (c_1_) and medium (c_2_) concentrations of the combination of Agarikon.1 and Agarikon Plus (AP + AG.1) likewise induced a significant apoptosis induction. The proportion of apoptotic cells after treatment with the highest (c_1_) concentration of Agarikon.1 could not be determined here either due to precipitate formation interfering with the assay. Significant increase in population of early apoptotic cells was observed after treatment with medium concentration (c_2_) of Agarikon.1 (50.31 ± 0.05%) and highest concentration of AP + AG.1 (45.98 ± 5.08) for 24 h with respect to control (5.23 ± 1.5%). Treatment with higher concentrations of Agarikon Plus result in about 15% of cells in early apoptosis. A statistically significant percentage of cells in late apoptosis relative to control (2.22 ± 1.22%) was noted only after treatment with the highest concentration of the extract mixture (6.94 ± 2.63%). As with HCT-116, treated SW620 cells did not exhibit statistically significant difference in the proportion of cells in necrosis compared to control cells.

### 2.3. In Vivo Analysis

#### 2.3.1. Survival and Tumor Volume Analysis

**Figure 3:**
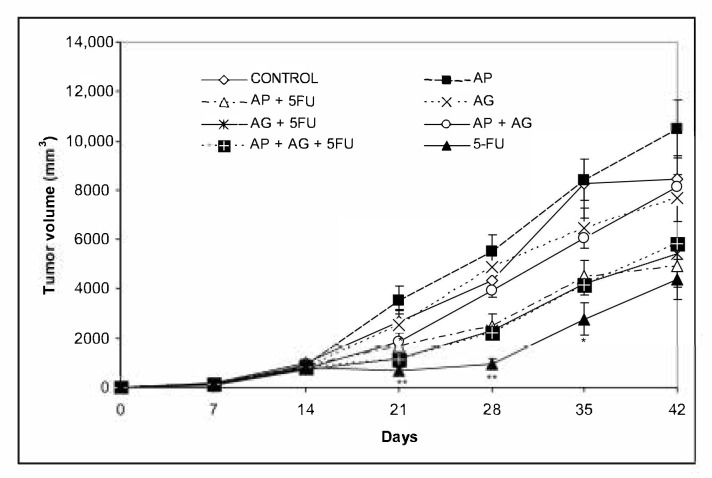
Changes in tumor volume of Balb/c mice after treatment with Agarikon Plus, Agarikon.1 and 5-fluorouracil in various combinations. Treatment started on day 14 after s.c. inoculation of CT26.WT (1 × 10^6^/mouse) cells. Volumes were determined once weekly for six weeks. * Significantly different (* *p* < 0.05; ** *p* < 0.01 in comparison with control group. Bars show means ± SEM.

Mice bearing CT26.WT tumors administered with various combinations Agarikon.1 (AG.1), Agarikon Plus (AP) or 5-fluorouracil for two weeks after tumor inoculation showed a significant increase in life span in most curative subsection groups (Table 3). In groups treated with mushroom extracts only, the best effect on survival was noted in group treated with AP + AG.1 (OS = 55.5%). The best overall survival in all groups was observed in group treated with AG.1 + 5-FU (OS = 87.5%), surpassing the one in group treated with 5-FU alone (OS = 77.7%). Importantly, in the preventive subsection, all animals treated with either AG.1 or AP were still alive (*n* = 10) on day 45 after tumor cell inoculation. The length of survival observation in this subsection was based according to literature, where the usual life span of 34 ± 6.2 days is observed in CT26.WT inoculated non-treated animals [30,31]. Tumor volume reduction with respect to control was observed in all treatment groups except in group treated with AP, while statistical significance was only noted in group treated with 5-FU (Figure 3).

#### 2.3.2. Effect on Macrophage Polarization

Tumor microenvironment is characterized by the prevalence of M2-like macrophages, tumor associated macrophages (TAMs). Macrophages in this activation state have important roles in immunosuppression, promoting cancer cell motility, as well as in promoting tumor angiogenesis and metastasis, while classically activated M1 macrophages have increased tumoricidal and microbiocidal abilities [32]. Amino acid metabolism is closely related to functional phenotype, and M1 macrophages are characterized by increased expression of inducible nitric oxide synthase (iNOS or NOS2) and nitric oxide (NO) production, which is critical for the tumoricidal activity of the immune system. iNOS catalyzes the conversion of L-arginine to NO and citrulline. M2 macrophages are characterized by high expression of Arg-1 (arginase-1), a cytosolic enzyme that metabolizes L-arginine to ornithine and polyamine. Polyamines are important for collagen production, cell proliferation, fibrosis, and other processes associated with tissue remodeling [33,34]. Therefore, nitric oxide (NO) and arginase 1 activity are important indicators of macrophage polarization. Our results indicate that treatment with various combinations of AP, AG.1 and 5-FU reduce arginase 1 activity as shown in Table 4. This decrease was most pronounced in AP + 5-FU (*p* < 0.001) and AG.1 (*p* < 0.05) groups. The levels of NO are elevated in all treatment groups in comparison with control, which is especially evident in AP + 5-FU and 5-FU groups, where these changes are statistically significant.

#### 2.3.3. Effect on Cytokine Levels

**Figure 4:**
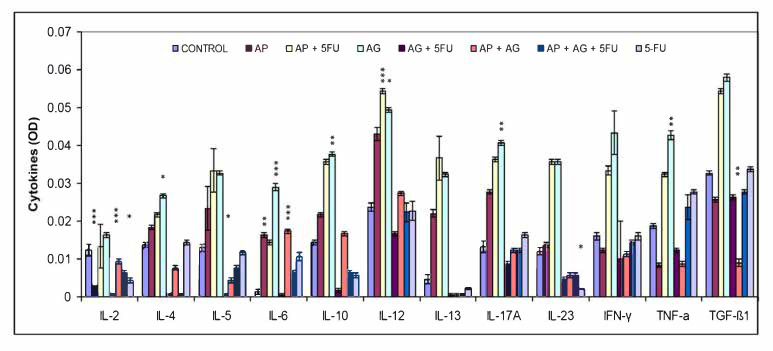
Effect of treatment with Agarikon Plus, Agarikon.1 and 5-fluorouracil in various combinations on cytokine levels in serum supernatant of mice bearing CT26.WT tumors. Mice were sacrificed on the 28th day and cytokine level in serum supernatant was measured by ELISA kit according to the manufacturer’s instructions (Multi-Analyte ELISArray kit, Qiagen) for mouse. * Significantly different (* *p* < 0.05; ** *p* < 0.01; *** *p* < 0.001) in comparison with control group. Data (mean ± SD) shown are from one representative experiment, with *n* = 3 per treatment group.

In addition to nitric oxide (NO) and oxygen radicals, M1 macrophages have Th1-orienting properties, which is characterized by secretion of high levels of classical proinflammatory cytokines such as interleukin (IL)-2, IL-6, IL-12, IL-23, TNF-α and IFN-γ. Besides glucocorticoids and prostaglandins, M2 functions are stimulated by Th2 citokines such as IL-4, IL-10 and IL-13 [35]. The change in the serum levels of Th1, Th2 and Th17 cytokines as a result of treatment is shown in Figure 4. In comparison to other cytokine levels, relatively lowest serum IL-2 levels were observed. In comparison with control group, a slight tendency of increase in IL-2 was found only for the group treated with AG.1. For most of the remaining cytokines (IL-4, IL-5, IL-6, IL-10, IL-12, IL-13, IL-17A, IL-23, IFN-γ, TNF-α, TGF-β1), in general, the highest levels of cytokines were measured in the groups treated with AP, AP + 5FU and AG.1, and in most of these cases a gradual increase in the concentration of these cytokines is: control < AP < AP + 5FU < AG.1. In the remaining four groups (AG.1 + 5FU, AP + AG.1, AP + AG.1 + 5FU and 5FU), the concentrations of these cytokines mostly fall, and are close to control levels.

#### 2.3.4. Antiangiogenic Effects

To grow and survive, as well as to maintain the process of metastatic spread, tumor cells need the proximity of blood vessels [36]. The onset of angiogenesis, i.e., the angiogenic switch, is often described as a relative increase in factors that stimulate this process, in relation to various inhibitors of angiogenesis or angiostatic factors. VEGF secretion is crucial in stimulating tumor angiogenesis, vascular homeostasis and early stages of vasculature development [37]. VEGF measurement is one of the standard methods for assessing the effectiveness of antiangiogenic therapies [38]. Our results show a tendency in reduction of VEGF concentration in all treatment groups, which reached statistical significance for groups treated with AP (*p* < 0.05), AP + AG.1 (*p* < 0.01) and AP + AG.1 + 5-FU (*p* < 0.001) (Table 5).

#### 2.3.5. Effects on Matrix Metalloproteinase Levels

Matrix metalloproteinases are zinc containing endopeptidases which influence various steps during metastatic process, such as angiogenesis, regulation of inflammation and metastatic niche maintenance. It is known that synthesis of MMPs by endothelial cells can have opposite effects on tumor angiogenesis, facilitating extracellular matrix degradation and new blood vessel formation, but also blocking angiogenesis by producing inhibitors of endothelial cell growth, such as angiostatin [39]. The concentration of MMP-2 determined in the serum samples of treated animals was increased in almost all groups compared to the control, and in the group treated with AG.1 + 5-FU this difference was statistically significant (*p* < 0.01). A slight decrease in the concentration of this protein was observed only in the group treated with AP (Table 6). Matrix metalloproteinase 9 (MMP-9) serum concentration showed an increase in certain groups compared to the control (treatment with AP, AG.1), while groups treated with AP + 5-FU, AG.1 + 5-FU, AP + AG.1 + 5-FU, and 5-FU observed a slight decrease in MMP-9 concentration relative to control, but without statistical significance. The difference between certain groups with elevated in comparison to those with decreased concentrations of MMP-9 was statistically significant, namely AP vs. AP + AG.1 + 5-FU (*p* < 0.05), AP vs. AG.1 + 5-FU (*p* < 0.01), AP vs. 5-FU (*p* < 0.05), AG.1 vs. AG.1 + 5-FU (*p* < 0.05), AG.1 vs. 5-FU (*p* < 0.05).

#### 2.3.6. Comet Assay

The effect of treatment on comet parameters, namely tail length, tail intensity and tail moment was expressed as fold change (FC) in comparison to control. The analysis of peripheral leukocytes shown that these parameters were elevated in all treatment groups compared to control and are statistically significant (Table 7). Likewise, comet assay parameters in hepatocytes of animals treated with AP + 5-FU, AG.1 + 5-FU, AP + AG.1, AP + AG.1 + 5-FU and 5-FU were elevated and significant (*p* < 0.001) (Table 8).

## 3. Discussion

Medicinal mushrooms have a large number of bioactive substances, and their indirect antitumor effects are primarily attributed to enhanced activation of the host immune system, which results from high content of various complex polysaccharides, of which β-glucans are the most studied. More recently, it has been found that various classes of compounds from medicinal mushrooms have significant direct antiproliferative or cytotoxic effects on tumors, mediated by inhibition of protein kinases and other signaling pathways (Wnt, EGFR, VEGFR, Jak/Stat, mTOR, Shh, TGF/Smad and others), DNA polymerases and DNA topoisomerases, as well as by modulation of G1/S and G2/M cell cycle checkpoints [18,40]). Our recent study has shown that the formulation Agarikon.1 can downregulate proteins essential in ribosome biogenesis and translation, which represents a fundamental process which is ubiquitously dysregulated during colorectal cancer progression [41].

Significant dose-dependent cytotoxicity observed in SW620 and HCT-116 cells is in line with a large body of research documenting antiproliferative effects of various mushroom species used in formulations Agarikon.1 and Agarikon Plus. Cytotoxic effects of many mushroom species are the result of apoptosis induction. Dose-dependent cytotoxicity of *Trametes versicolor* extract was established on four human breast cancer cell lines, including MCF-7, where the main mechanism is an induction of cell death (apoptosis) due to increased p53 expression paralleled by decreased expression of antiapoptotic Bcl-2 protein [42]. Polysaccharopeptide PSP is the most well-studied substance from *Trametes versicolor*, and has been confirmed in several studies to act selectively in cancer cells, with minimal effects on healthy cells [43]. An extract of the same species dose-dependently inhibits the proliferation of HeLa and HepG2 cells [44]. The activity of the enzymes laccase, peroxidase and glutathione reductase was determined in the extract, and the antitumor activity is a consequence of the presence of natural quinone substances in the extract which are formed by the activity of these enzymes on the lignin substrate. Tumor cells, unlike normal ones, have a high content of NAD(P)H-quinone oxidoreductase (DT-diaphorase), which converts quinones into substances that are toxic to the cell. This is why the extracts of these species are considered to be toxic specifically to tumor cells. *Ganoderma lucidum* is one of the most studied species of medicinal mushrooms, and its dose-dependent induction of apoptosis in SW620 metastatic colorectal cancer cells is the consequence of caspase-3, Bax and p53 activation, with simultaneous inhibition of Bcl-2 expression at mRNA and XIAP at the protein level [45]. The bioactive protein C91-3 apoptotic protein 24,414 was isolated from *Lentinus edodes*, which has a dose-dependent effect on reducing proliferation and increasing the proportion of A549 lung adenocarcinoma cells in apoptosis [46]. Antiproliferative and proapoptotic activity of *Agaricus blazei* extract results from strong inhibition of telomerase activity, induction of cytochrome release, caspase activation, and regulation of Bcl-2 synthesis [47].

Mild (AP) to no cytotoxicity (AG.1) was observed in healthy human fibroblast cells WI-38, and regenerative activity was also observed with higher concentrations of Agarikon Plus and Agarikon.1 (Figure 1). Such results are in agreement with some studies that have shown the proliferative activity of certain medicinal mushroom extracts on fibroblasts, which can result in faster wound healing [48]. Certain components of extracts, such as caffeic acid, can have an antioxidant effect on healthy cells and a prooxidative effect on tumor cells, leading to oxidative DNA damage and induction of cell death (apoptosis) in tumor cells [49].

Some medicinal mushrooms and their components are an example of potential drugs or medical products with a pleiotropic action, i.e., simultaneously affect multiple pharmacological targets or causing their inhibition (multitarget agents or multitarget inhibitors), which is also a property of many other natural products and their isolates, such as soy isoflavones and other legumes, turmeric from various species of the genus *Curcuma* from the ginger family, resveratrol and many other natural (poly)phenols and their subgroups, such as epigallocatechin-3-gallate (EGCG) from green tea. In the treatment of complex pathologies such as cancer, such an approach of multiple pharmacological targets is necessary, which is further emphasized by the fact that pharmacological approaches to cancer treatment which involve one or several targets are usually very short-lived and yield weaker treatment outcomes [50]. In this regard, the use of combination of several medicinal mushroom species is also based on the observation that mixtures of polysaccharides from several species of certain medicinal mushrooms can in some cases achieve both cumulative immunomodulatory and other antitumor effects. Synergistic immunostimulatory effects may result from a cumulative activation of multiple receptor types on immune cells, such as dectin receptor-1 and various Toll-like receptors, which can lead to stronger expression and synthesis of different immunostimulatory cytokines, while synergistic direct antitumor effects can result from the activation of multiple cancer-inhibitory pathways and effects [18,51].

Recently, new pharmacological mechanisms of action of various chemotherapeutics that have been used for a long time in standard therapies for various forms of cancer have been discovered. One of the main mechanisms observed in certain cytotoxic chemotherapies is their immunostimulatory effect. Although chemotherapeutics, when administered in higher doses, with the main goal of reducing tumor mass (cytotoxic effect) have primarily an immunosuppressive effect due to their toxicity to the bone marrow, their use in lower doses may lead to the opposite effect [22,52]. Therefore, in our study, 5-fluorouracil was dosed metronomically (metronomic scheduling protocol), which implies lower doses than those used during the classic dosing of this chemotherapeutic. Such dosing, in addition to immunostimulatory, also reduces the toxic effects of chemotherapeutics, significantly affects the inhibition of angiogenesis and reduces the possibility of developing drug resistance [53]. For this purpose, such dosing is often used in the clinic where a higher dose is given for the first four days, after which half of that dose every other day for four days is administered, and we also applied this scheme in our study [54]. By using chemotherapeutics in such so-called suboptimal doses, it is assumed that immunosuppressive cells are preferentially destroyed over effector immune cells. In this way, the last step of immune editing of the tumor, which includes immune escape, could potentially be prevented, and more significant effects of various tumor immunotherapies can be achieved, where the use of fungal substances is conceptually closest to active nonspecific immunotherapy according to standard classification.

The significance of the CT26.WT syngeneic model in tumor immunology research was also confirmed by the fact that the increased activity of tumor associated macrophages (TAMs) in Balb/c mice resulted in increased tumor growth, angiogenesis and metastasis [55]. In addition to the use of a very aggressive and undifferentiated colon cancer cell line CT26.WT, treatment with Agarikon.1 and Agarikon Plus, alone or in combination with 5-fluorouracil was started only when the tumor volume averaged 800–1000 mm^3^, which is a very late disease model [56]. For comparison, early models are defined by the start of treatment often as soon as on the next or only a few days after tumor inoculation, when the average values of tumor volumes are 40–60 mm^3^.

The idea that the immune system models tumor immunogenicity is the basis of the cancer immunoediting hypothesis, which means that the host’s immune system can have a protective or promoting effect on the tumor [57]. Immune editing is a process that occurs only after malignant transformation has already occurred and is an extrinsic tumor suppressor mechanism that is activated after the failure of intrinsic tumor suppressor mechanisms, such as DNA repair. Because various groups of medicinal fungal substances, primarily polysaccharides and polysaccharide and protein complexes (polysaccharopeptides), have numerous immunostimulatory effects, this may also explain 100% survival of animals up to 45 days after tumor cell inoculation in a preventive subsection treated with Agarikon.1 or Agarikon Plus (Table 3). Since we used an induced rather than a spontaneous tumor, it can be assumed that treatment with Agarikon.1 or Agarikon Plus leads to increased immune cell activation, which can therefore prolong the equilibrium phase, which is the second phase of the tumor immune response according to cancer immunoediting hypothesis. Namely, it is known that the role of the acquired immune system is particularly important in the equilibrium phase, with key roles of interleukin-12 (IL-12), IFN-γ and CD4^+^ and CD8^+^ T lymphocytes [58]. Accordingly, studies confirm that in the advanced stages of the tumor, the number of neutrophils and macrophages is increased, and the number of T and B lymphocytes is reduced [59]. Therefore, one of the key immunotherapeutic goals in advanced tumors could be to maintain immune balance for as long as possible. In solid tumors, one of the key obstacles, therefore, in addition to the reduced effectiveness of antitumor effector cells, is the effect of the tumor microenvironment, which inhibits the effect of activated cells in situ, i.e., in the tumor tissue itself. Although a study of 6 tumor models showed that Ki-67 proliferation indices in weakly immunogenic and highly immunogenic (including CT26) tumors were similar, more immunogenic tumors showed slower growth in immunocompetent mice than less immunogenic ones [56]. This indicates the differential significance of potential inhibition of tumor growth by activating the immune system versus classical cytotoxic chemotherapy, which has the most pronounced effect on rapidly proliferating tumor cells. In our study, inhibition of tumor growth was most pronounced with the use of 5-FU, while the reduction was less pronounced with the use of AP, AG.1 alone or in combination (Figure 3). In none of the groups was a synergistic effect of 5-FU with AG.1 or AP on a more pronounced reduction in tumor volume observed, compared to 5-FU alone. However, the best survival was recorded in the group treated with AP + 5FU, although the tumor volume was higher in this group compared to the treatment with 5-FU (Figure 3, Table 3), which is consistent with the data that survival in CRC does not show a correlation with volume, namely the size of the primary tumor [60,61]. Additionally, survival was better in the AG.1-treated group alone (OS = 50%; OS, overall survival) compared to the AG + 5FU-treated group (OS = 33%), although tumor volume was lower in the latter group. Altogether, Pearson’s correlation coefficient between final tumor volume and overall survival (OS) was *r* = −0.533, indicating a moderate negative correlation. In a study on the CT26.WT model, Wu et al. (2016) noted a significant difference in survival between untreated and Balb/c mice treated with 5-fluorouracil (40 mg/kg), starting at day 12 after tumor cell inoculation [62]. However, although multiple cycles of chemotherapy resulted in a reduced increase in tumor volume, compared to one cycle of chemotherapy, no significant difference in improvement in overall survival (OS) was observed between mice that underwent one (C1) compared to mice that underwent two (C2), three (C3), or four (C4) cycles of 5-fluorouracil chemotherapy. This was associated with only a short-term improved immune response after a single cycle of chemotherapy, as the percentage of tumor infiltrating CD8^+^ lymphocytes was increased after cycle 1 of 5-FU (C1) but not after cycle 3 (C3). Repeated cycles of chemotherapy further reduced the number of CD4^+^ lymphocytes as well as B-lymphocytes. The same study also noted that tumor growth was inhibited during chemotherapy itself, and accelerated between chemotherapy cycles, which is also consistent with the observed dynamics of tumor growth in the groups that included 5-FU administration in our study (Figure 3). Finally, this study confirms the independence of survival parameters relative to tumor volume on the CT26 model, which is consistent with clinical data.

In addition to the Th1 and Th2 immune responses which are analogous to those mediated by M1 and M2 macrophages, respectively, Th17 are the third major subtype of Th cells that produce IL-17, IL-6, IL-8, and IL-26 and promote inflammation and granulocyte activity [63]. IL-23 is a cytokine that is essential as a key regulator of IL-17 expression, and is necessary for the induction, survival, and expansion of Th17 cells [64]. Th17 cells are characterized by their plasticity, and can, depending on the tumor microenvironment, have protumor effects, largely by stimulating tumor angiogenesis, while in other cases IL-17 stimulates IFN-γ^+^ effector T lymphocytes and NK cells in the tumor microenvironment, leading to increased antitumor immunity [65]. Adaptive transmission has shown that Th17 cells have a superior antitumor effect over Th1 and Th2 cells, and are considered long-term memory cells that mediate long-term antitumor immunity due to their longer retention in the tumor microenvironment. Due to the above, it has been suggested that Th17 and Th1 cells are phenotypically and functionally related in the tumor microenvironment [66]. Macrophage Toll-like receptors (TLRs) are one of several groups of receptors involved in promoting non-specific immunity. Fungal polysaccharides can stimulate TLR signaling and lymphocytes and macrophages via TLR4 receptors, while TLR2, which is thought to be primarily a receptor for bacterial lipoproteins, activates dendritic cells, T lymphocytes, and NK cells [67]. Induction of cytokines such as TNF-α and MIP-2 (CXCL2) requires signals from TLR2 and TLR6 [68]. In order to assess the polarization of the immune response, the proportions of Th1/Th2/Th17 cytokines in the serum of Balb/c mice were quantified after treatment with AP, AG.1, 5-FU and their mutual combinations (Figure 4). An increase in Th1 cytokines compared to the control group was observed especially in the groups treated with AP (IL-6, IL-12), AP + 5FU and AG.1 (IL-6, IL-12, IFN-γ, TNF-α). A significant increase in the Th17 cytokines IL-17α and IL-23, as well as IL-6 in AG.1, was also observed in the groups treated with AP + 5FU and AG.1. IL-12, the main mediator and activator of innate immune cells, in addition to macrophages, also stimulates NK cells, which leads to the synthesis of IFN-γ, which has a strong antiproliferative role. Cytokines have pleiotropic properties and often, depending on the (microenvironmental) context, can act to stimulate or suppress tumor development. Thus, TNF-α is known to be a proinflammatory cytokine that can stimulate tumor growth, angiogenesis and metastasis, through activation of NF-κB, VEGF and MMPs, while in other cases its antitumor role mediated by tumor stroma destruction predominates by stimulating cytotoxic T lymphocyte and macrophage tumor infiltration and dendritic cell activation, which can lead to strong regression of malignant tumors [69,70]. TGF-β is also an example of a differentiating cytokine, which has contextually different roles, and most often in the early tumor stages has proapoptotic or antitumor, while in the later tumor stages it can stimulate epithelial-mesenchymal transition [71]. Given that a significant increase in TGF-β1 was also observed in the groups treated with AP + 5FU and AG.1 which also have a significant increase in Th1 cytokine levels, it can be concluded that TGF-β had antiproliferative properties [72]. In the same groups, the highest increase in IL-10 was observed, which is important for the normal function of helper CD4^+^ lymphocytes, CD4^+^ lymphocyte-mediated immune response, and suppression of tumor-mediated inflammation. Thus IL-10 represents a key antitumor cytokine [73]. Although AP or AG.1 alone showed significant effects on the secretion of different cytokine subgroups, no cumulative effect on elevated cytokine levels was observed in the group treated with AP + AG.1, which may indicate a competition of AP and AG.1 components for immune cell receptors.

In addition to the cytokine profile, the key difference between M1 and M2 macrophages is in their amino acid metabolism, which plays key roles in their functions. M1 macrophages are characterized by increased expression of iNOS (NOS2) and production of NO, which is an important effector of their microbiocidal and tumoricidal activity. The roles of NO in tumor development and progression are multiple; at low concentrations (<100 nM) it stimulates proliferation and angiogenesis, at medium (100–500 nM) increased invasiveness, metastasis, and suppression of apoptosis, while at higher (>500 nM) it stimulates DNA damage, oxidative stress, cytotoxicity due to peroxynitrite production and apoptosis [74]. Arginase 1, which in addition to M2 macrophages in the tumor microenvironment is classically secreted by myeloid-derived suppressor cells (MDSC), has various immunosuppressive roles. In addition to its suppressive effect on NO, arginase 1 has been shown to inhibit T lymphocyte receptor (TCR) expression in the tumor microenvironment and thus obstruct specific antigen-dependent responses. Arginase 1, like arginase 2, hydrolyzes L-arginine to urea and L-ornithine, which is the main substrate for polyamine formation. Polyamines are necessary for cell cycle and tumor progression [75]. High arginase activity has been reported in patients with gastric, colon, breast, and lung cancers. By analyzing the obtained results of NO and arginase-1 concentrations in the samples, it has been proven that the tested preparations and their combinations have a significant effect on macrophage polarization. Thus, in all treated groups, an increased concentration of NO was recorded compared to control, and is present in cytotoxic concentrations (higher than 500 nM) (Table 4). The most significant increase in NO concentration was recorded in the groups treated with AP + 5-FU, AP + AG.1 and 5-FU, which are also the three groups with the best survival. On the other hand, the activity of arginase-1, one of the main markers of M2, as well as TAM macrophages, was significantly reduced in all treated groups compared to the untreated group, and has reached statistical significance in the groups treated with AP + 5-FU, AG.1 and 5-FU, which showed the best survival. Based on the above data, we conclude that macrophage polarization from tumor (M2) to antitumor (M1) phenotype occurs in all treated groups. Furthermore, repolarization of TAM in M1 macrophages results in other, non-immune effects mediated by macrophages, such as a reduction in levels of proangiogenic factors such as VEGF, MMP-2, and MMP-9. The effect of polarization of tumor-associated macrophages (TAM) from immunosuppressive to activated i.e., tumoricidal M1 macrophage phenotype has been recognized as one of the main antitumor effects of β-glucans [76]. Bioactive polysaccharides from medicinal mushrooms have previously been thought to stimulate an immune response independent of T lymphocytes. It was subsequently found that β-glucans, after being processed into low molecular weight carbohydrates, bind to MHC-II (major histocompatibility complex class II) within dendritic cells, which can lead to direct activation of CD4^+^ lymphocytes and Th1 responses [77]. Furthermore, it is known that when administered at certain doses and intervals (chemotherapeutic protocol), some chemotherapeutics may have targeted (acting on tumor cells) and non-targeted (acting on immune cells) immunostimulatory effects [22].

Proteolytic degradation of the basement membrane and surrounding extracellular matrix (ECM) is a necessary process in tumor metastasis and spread. Matrix metalloproteinases, such as MMP-2 and MMP-9, in addition to degrading most ECM proteins, also affect the activity of other proteases, growth factors, cytokines, and various cell surface ligands and receptors [78]. Although in most tumors reduced levels of MMP-2 and MMP-9 correlate with better prognosis, and reduced invasiveness and metastasis, the role of matrix metalloproteinases is pleiotropic [79]. This also explains the fact that MMP inhibitors developed in the clinic have proved unsuccessful [80]. MMP-3, -7, -9, and -12 in addition to plasmin have been found to participate in the release of angiostatin from plasminogen thereby reducing endothelial cell proliferation and migration [81]. MMP-9 cleaves and activates antiangiogenic precursors angiostatin, endostatin, and tumstatin by its protease activity, which all have an inhibitory effect on tumor growth and vascular tumor density [82]. Tumor stage also has a significant effect on the effects of matrix metalloproteinases. Thus, in advanced colon cancer (CRC), high levels of MMP-12 have been associated with better prognosis, while increased levels of TIMP, the negative tissue regulator of MMP, correlated with a negative prognosis in that cancer [83]. This dual role of matrix metalloproteinases is also visible in our results, where in all treated groups, except those treated with AP, an increased concentration of MMP-2 was found compared to the control (Table 6). The concentration of MMP-9, on the other hand, was increased compared to the untreated group in the groups treated with AP or AG.1, and slightly decreased in the groups treated with 5-FU alone or in combination with AP or AG.1 (Table 6). In general, a very weak correlation was found between MMP-2 and MMP-9 levels and survival and tumor volume.

Angiogenesis is a key process necessary for tumor growth and metastasis, and is defined as formation of new blood vessels from the existing microvasculature [84]. Because VEGF-VEGFR is the major signaling pathway for tumor vascular formation and remodeling, monitoring changes in VEGF levels is one of the standard methods for assessing antiangiogenic therapies [38]. In a number of tumor types, including CRC, VEGF has been recognized as a negative prognostic factor [85]. The use of antiangiogenic therapies may improve the outcomes of conventional antitumor therapies due to increased tumor specificity and reduced development of tumor cell resistance [86]. The obtained results show a significant decrease in VEGF concentration in all treated groups compared to the control (Table 5). These results are in line with previous research on the effects of certain types of medicinal mushrooms. Cao and Lin (2006) found that the Gl-PP polysaccharopeptide isolated from *Ganoderma lucidum* has antiangiogenic properties resulting from direct apoptosis of HUVEC (human umbilical vein endothelial cells) cells, as well as a decrease in antiapoptotic Bcl-2 expression and an increase in Bax expression in these cells [87]. Since VEGF levels were also reduced in the groups where elevated concentrations of MMP-2 and MMP-9 were recorded, this further indicates the previously mentioned dual role of matrix metalloproteinases, which likely leads to increased production of antiangiogenic factors that contribute to reduced tumor aggressiveness and an increased survival. Our results of the cumulative effect of metronomically dosed administration of 5-fluorouracil and immunotherapeutic action of AG and AP.1 on the reduction of VEGF levels also confirm the results of previous studies. Wada et al. (2007) confirmed that the combination of interferon-α immunotherapy and metronomically dosed 5-fluorouracil has a cumulative effect on reducing angiogenesis and tumor growth in a mouse model [88].

Due to its sensitivity, the comet test or single cell gel electrophoresis (SCGE) in alkaline conditions is a suitable method for assessing different types of DNA damage, such as single-stranded and double-stranded DNA breaks, apurine and apyrimidine sites, and DNA repair [89]. Thus, by measuring cytotoxic or genotoxic effects using a comet test on tumor cells, it is possible to determine the therapeutic effectiveness, while on healthy cells it is a standard method of determining the potential adverse effects of therapies. In our research, we performed comet assay analysis on peripheral leukocytes and hepatocytes, in order to establish potential genotoxic effects of treatment on healthy tissues. Our results of the alkaline comet test on peripheral blood leukocytes and hepatocytes indicate a slight increase in all parameters of the comet test, which is statistically significant almost everywhere due to the large number of samples (Table 7 and Table 8). Since a large number of samples are used in the analysis of comet test results, where each cell represents one statistical sample, the possibility of exaggerating statistical significance as a measure of biological effect is particularly emphasized. Therefore, in order to facilitate the assessment of the biological effect, i.e., the significance of comet test results as a measure of cytotoxicity or genotoxicity, we compared absolute values between individual parameters of a particular treated group in comparison with negative control, as described by [90]. A single substance may have cytotoxic or genotoxic effects depending on the dose. A genotoxic substance can also have mutagenic effects, making it a carcinogenic substance. As a reliable method of distinguishing the cytotoxicity and genotoxicity of an individual substance using a comet test, the relative ratio (FC, fold change) of individual parameters is used, most simply the tail moment, which is the product of the remaining two parameters [91]. Daza et al. (2004) compared the tail moment relative ratios of cytotoxic (cordycepin, fluorodeoxyuridine, puromycin) and known genotoxic (camptothecin, actinomycin C) substances on leukocyte cells. Mean tail moment parameter of cytotoxic substances ranged from 0.8–1.07, while genotoxic substances were characterized by significantly higher tail moments, which were 5.16–8.27 for actinomycin C and 6.30–12.13 for camptothecin, depending on the applied concentration of the substance [92]. In our study, we found that in all treated groups, the obtained tail moment ratios exclude genotoxic effect of the tested preparations Agarikon Plus, Agarikon.1, 5-fluorouracil, and their mutual combinations. Our data for 5-fluorouracil are in agreement with existing data on its tail-moment ratios when this drug was used in various chemotherapeutic protocols [93]. The non-genotoxic effects of tested mushroom extracts is likewise in line with previous data which confirm that several polysaccharides in clinical use have been shown to lack long-term toxicity, i.e., genotoxicity or mutagenicity, and can mitigate the genotoxic effects of chemotherapy and radiotherapy [94].

In conclusion, our data confirms antitumor effectiveness of blended medicinal mushroom preparations Agarikon.1 and Agarikon Plus in the advanced colorectal cancer mouse model, which is a good basis for further translational research. Furthermore, our results support the usefulness of such preparations use in enhancement of the effectiveness of standard cytotoxic chemotherapies by improving their immunomodulating, antiangiogenic and antimetastatic properties.

## 4. Materials and Methods

### 4.1. Tested Substances

Medicinal mushroom extract mixture Agarikon.1 (LOT:1100517) was provided by Dr Myko San—Health from Mushrooms Co, Croatia. This preparation has been registered by the Ministry of Health and Social Welfare of the Republic of Croatia as a dietary supplement (registration number MZ 0813411210) [20]. It is produced from a hot water extract which is precipitated with ethanol and subsequently freeze-dried. This tablet preparation contains a mixture of *Lentinus edodes*, *Ganoderma lucidum*, *Agaricus brasiliensis* (*blazei* ss. Heinem.), *Grifola frondosa*, *Pleurotus ostreatus* and *Trametes versicolor* medicinal mushroom species, in equal amounts, i.e., 125 mg each per tablet. One 1000 mg tablet therefore contains 750 mg of mushroom polysaccharides per tablet, combined with excipients such as inulin, talc, magnesium stearate and silica. Agarikon Plus (LOT:BXSM0901), obtained from the same manufacturer, is a proprietary liquid extract mixture of 10 mushroom species, including *Lentinus edodes*, *Ganoderma lucidum*, *Grifola frondosa*, *Trametes versicolor*, *Agaricus brasiliensis* (*blazei* ss. Heinem.), *Meripilus giganteus*, *Pleurotus ostreatus*, *Fomes fomentarius*, *Phellinus linteus*, and *Tricholoma matsutake* in equal amounts. As described before [27], 50 g of dried mushroom fruiting bodies are extracted in 1 L of boiling water for 24 h. Insoluble matter is removed by forcing the solution (in suspension form) through a filter press, and then concentrated 4-fold.

5-fluorouracil (manufacturer: Sandoz, Holtzkirchen, Germany), the antimetabolite class chemotherapy drug was supplied at a concentration of 50 mg/mL in sterile aqueous solution, pH 8.6 to 9.0, and stored at 4 °C in aluminum covered containers. Immediately prior to use, it was diluted in sterile distilled water.

#### 4.1.1. Determination of Dry Matter and Soluble Polysaccharides Content

To measure dry matter content, we used the Association of Official Analytical Chemists method [95]. The material was in an oven at 105 °C until constant weight was achieved. The moisture content was determined by the weight difference before and after drying, whereas the dry matter was the ratio of the final to the initial sample weight percentage.

The soluble polysaccharides content was determined according to a modified method of Wei et al. [96]. Briefly, liquid mushroom extracts were centrifuged (10,000 rpm for 15 min at 20 °C) to collect the supernatant, which was subsequently concentrated by vacuum evaporation until approximately 35° Brix was reached. The obtained concentrate was precipitated by the addition of two volumes of ethanol to a final concentration of 75% (*v/v*). The precipitates collected by centrifugation (10,000 rpm for 15 min at 20 °C) were solubilized in deionized water and lyophilized to obtain the crude polysaccharides. The tablet preparations were firstly ground to obtain a fine powder and were then dissolved in distilled water as described above in order to obtain liquid samples. The content of soluble polysaccharides was determined according to the previously described procedure. Polysaccharide yield was expressed as milligrams per gram of dry matter of sample.

#### 4.1.2. Determination of Polyphenolic Compounds

Total phenol content (TPC) of medicinal mushroom preparations was determined spectrophotometrically according to a modified Lachman method [97]. To determine total flavonoid content (TFC), tested compounds were precipitated using formaldehyde, which reacts with C-6 or C-8 on 5,7-dihydroxy flavonoids. The condensed products of these reactions were removed by filtration and remaining nonflavonoid phenols were determined according to the previously mentioned procedure for the determination of TPC. Flavonoid content was calculated as the difference between total phenol and nonflavonoid content. Gallic acid was used as the standard and the results were expressed as milligrams of gallic acid equivalents (GAE) per liter. All measurements were performed in triplicate.

### 4.2. In Vitro Experiment

#### 4.2.1. Cells and Culture Conditions

Human tumor cell lines HCT-116 (colorectal carinoma), SW620 (metastatic colorectal adenocarcinoma) and WI-38 (immortalized human lung fibroblast) were obtained from US American Type Culture Collection (ATCC, Manassas, VA, USA). Cells were cultured in a liquid nutrient medium (Dulbecco’s Modified Eagle Medium, LONZA, Basel, Switzerland) supplemented with 10% fetal bovine serum (FBS, Gibco, Grand Island, NY, USA), 2 mM L-glutamine (GIBCO, Grand Island, NY, USA), 100 μg/mL penicillin, and 100 μg/mL streptomycin (LONZA, Basel, Switzerland), in an incubator (NÜVE, Ankara, Turkey), at a humid atmosphere with 5% CO_2_ at 37 °C. Trypsin/EDTA Solution (Lonza, Basel, Switzerland), was used for cell passaging and cell detachment.

#### 4.2.2. Cytotoxicity Assay

Adherent cells were seeded on 96-well microtiter plates (Falcon, New York, NY, USA) at a seeding density of 5000 cells per well, added in 150 μL of cell suspension in a nutrient medium. 24 h after seeding, HCT-116 and SW620 cells were treated with Agarikon.1 or Agarikon Plus. Agarikon.1 was applied diluted, used a dilution series of 10–100,000, indicating a concentration range from 13.32 μg/mL to 1.332 ng/mL, while the same dilution series for Agarikon Plus indicates a concentration range from 6.4 μg/mL to 0.64 ng/mL. WI-38 cells were treated with both preparations using a dilution series of 2–20,000, indicating a concentration range from 32 μg/mL to 3.2 ng/mL for Agarikon.1 and from 66.6 μg/mL to 6.6 ng/mL. Various concentration ranges are based on the noted IC_50_ values for these cell lines. Incubation of the cells with the compounds were performed for 72 h in a 5% CO_2_, in humid atmosphere at 37 °C. After incubation, nutrient medium was removed and 40 μL of theMTTreagent (3-(4-dimethylthiazol-2-yl)-2,5-diphenyltetrazole bromide (Invitrogen, Carlsbad, CA, USA) was added to each well. 3 h after incubation, 160 μL of DMSO (VWR CHEMICALS, Radnor, PA, USA) were added to each well to dissolve the crystals of purple formazane. The absorbance was measured at 570 nm (TECAN SUNRISE Microtiter plate reader, Männedorf, Switzerland). The experimentally measured optical densities (OD) were converted to the PG (percentage of growth), using the formulas according to the NIH (National Institute of Health) protocol. The IC_50_ values (concentration causing 50% cell growth inhibition) for each compound were calculated by regression analysis of the survival count curve of the concentrations of the tested compounds. The results were processed in Excel 2010 (Microsoft, Redmond, WA, USA).

#### 4.2.3. Apoptosis Detection by Flow Cytometry

A commercial ApoAlert Annexin V kit (Clontech, Mountain View, CA, USA) containing annexin V-FITC, propidium iodide, and binding buffer was used to analyze cell death by flow cytometry. For the purposes of the experiment, 500,000 cells per well were seeded on a 24-well cell culture plates, and the cells were treated in three concentrations (dilutions of 100–10,000) with Agarikon Plus and Agarikon.1 preparations, as well as with their mixture with dry matter ratio of 1:1 and incubated for 24 h. After incubation, the medium was removed, the wells were washed with 0.025% trypsin for 5–10 min, and then transferred to tubes and centrifuged for 5–10 min at 1500 rpm. The supernatant was discarded while the cells were washed 2 times in cold FBS-PBS. The cells were then resuspended in binding buffer, 5 μL of Annexin V and 10 μL of propidium iodide were added and incubated for 15 min at room temperature in the dark. 400 μL of binding buffer was added afterwards to each tube, and cells were analyzed on a flow cytometer (BD Biosciences LSR II) fitted with a 488 nm argon laser. In each sample, 10,000 cells were sorted. The data were analyzed using CellQuest software. The cells were sorted into intact cells (Annexin V−PI−), early apoptotic cells (Annexin V+PI−), late apoptotic cells (Annexin V+PI+), and necrotic cells (Annexin V−PI+).

### 4.3. In Vivo Experiment

#### 4.3.1. Animals

Male Balb/c mice, approximately 2 months old, weighing 20–25 g were obtained from the Rudjer Boskovic Institute, Facility for laboratory animals, Zagreb. Animals were kept under conventional housing conditions and were maintained on a pellet diet (Standard Diet 4RF 21 GLP certificate, Mucedola, Italy), given water ad libitum, and subjected to an equal 12-h light/dark cycle in accordance with institutional guidelines. Experimental groups comprised 10 mice each. Animal studies were approved by the University of Zagreb, Department of Biology Ethics Committee (approval code: 251-58-10617-16-14) and performed in compliance with the guidelines in force in the Republic of Croatia (the Croatian Animal Welfare Law (NN, 135/2006 and 37/2013)) and according to the European Directive 2010/63/EU.

#### 4.3.2. Tumor Cells

CT26.WT (ATCC^®^ CRL-2638™) was obtained from American Type Culture Collection (ATCC). This is an *N*-nitroso-*N*-methylurethane-(NNMU) induced murine colorectal cell line syngeneic with Balb/c mice. This cell line shares molecular features with sporadic, aggressive, undifferentiated (stage IV), therapy-refractory human colorectal carcinoma cells, it is easily implanted and metastasizes readily [98]. The cells were propagated and subcultured in accordance to the distributor’s protocol, i.e., they were grown in RPMI-1640 medium with 10% FBS, penicillin (100 U/mL) and streptomycin (100 μg/mL) and maintained at 37 °C with 5% CO_2_ in a humidified atmosphere. After harvesting and preparation of cells, their total number and viability were determined by counting in a Neubauer chamber using Trypan Blue Dye, and was always found to be at least 95%.

#### 4.3.3. Experimental Design

Prior to treatment animals were divided into three study groups. The mice in the group study 1 (curative subsection) were injected subcutaneously in the right flank with 1 × 10^6^ viable CT26.WT cells in 100 µL of sterile PBS on day 0. Treatment of animals with tumors was started when the tumor was developed in 100% of the animals with a palpable solid tumor mass (≥700 mm^3^, 14 days post-implantation), which is a highly advanced stage of the tumor. On day 14, mice were randomly divided into 8 groups (*n* = 10 mice/group) and treated with 10,400 mg/kg of Agarikon Plus (AP) diluted in PBS, 1200 mg/kg of Agarikon.1 (AG.1) diluted in PBS by oral gavage during 14 days continuously, and with 5-fluorouracil (5-FU) intraperitoneally (30 mg/kg on days 1–4 and 15 mg/kg on 6, 8, 10 and 12 day of treatment). The treatment groups were treated with the following combinations of tested substances: AP, AP + 5-FU, AG.1, AG.1 + 5-FU, AP + AG.1, AP + AG.1 + 5-FU, 5-FU. The control was given only saline by oral gavage. 5-FU was administered metronomically, and for both preparations the doses were calculated by interspecies allometric scaling [99]. The basis for the dose calculation of Agarikon.1 and Agarikon Plus was the recommended daily dose of these dietary supplement used by patients. The survival was monitored until day 55 after tumor inoculation, after which the remaining animals were euthanized.

In the second study group, mice were treated in the same manner as the first, but the animals (*n* = 10 per group) were euthanized on day 28 after tumor cell inoculation, for the analysis of macrophage polarization by nitric oxide (NO) and arginase I activity assay, as well as for the comet assay. Serum supernatant was used for determination of Th1, Th2 and Th17 cytokines as well as VEGF and MMP-2 and MMP-9 concentrations. Third study group (preventive subsection) comprised two groups (*n* = 10 mice/group) which were treated with Agarikon.1 (1200 mg/kg) or Agarikon Plus (10,400 mg/kg) intragastrically for one week before and one week after tumor cell inoculation (1 × 10^6^ CT26.WT cells). The survival in these groups was monitored until day 45 after tumor cell inoculation, after which the remaining animals were euthanized. In this manner, mice in both curative (first study group) and preventive (third study) groups received treatment during equal time period.

#### 4.3.4. Survival and Tumor Volume Analysis

Animal life span was evaluated by daily surveillance of spontaneous death or by selective euthanasia of animals showing signs of pain and suffering according to established criteria. Kaplan–Meier statistical analysis (log rank statistics) as well as overall survival were utilized to compare survival. Analyses were performed using MedCalc (Version 19.1.3) and the significance level was 5% (*p* < 0.05). In order to analyze the inhibitory effects of tested substances on tumor growth, tumor length (L) and width (W) was measured and tumor volume (mm^3^) was calculated as [V = (L × W^2^)/2]. Data was analysed by Kruskal–Wallis ANOVA.

#### 4.3.5. Preparation of Spleen Macrophages

Spleens were dissected from abdominal cavity and filtered through a 40 μm nylon strainer. Cell suspensions were diluted 1:2 with DMEM and layered on Lymphoprep (Nyegard, Oslo, Norway) and centrifuged at 400× *g* for 20 min at room temperature. The mononuclear cell layer was collected after adherence within 3 h and washed twice with medium. The cell concentration was adjusted to 1.0 × 10^6^ cells/mL with 0.1 μg/mL LPS in DMEM containing 10% FCS at 37 °C in an atmosphere of 5% CO_2_. Adherent cells were separated from nonadherent and incubated in fresh culture medium for 24 h. Supernatant and adherent cells obtained by scraping the plastic surface with a rubber policeman, were used for NO and arginase 1 analysis. Each sample was analysed in triplicate.

#### 4.3.6. NO Production

NO concentration in treated and control groups were measured with a colorimetric assay kit (Griess Reagent System, Catalog Number G2930, Promega, Madison, WI, USA) according to the Griess reaction. Macrophages cell layer in a 96-well plate were incubated with 0.1 μg/mL LPS in DMEM containing 10% FCS at 37 °C in an atmosphere of 5% CO_2_. The nitrite concentration in cell supernatants, taken as a measure of NO production, was determined at an interval of 24 h. It was detected in individual cell-free samples (50 mL) incubated 10 min at ambient temperature with an aliquot of the Griess reagent (1% sulphanilamide in 5% phosphoric acid and 0.1% naphthylethylenediamine dihydrochloride in water). The absorbance at 540 nm was recorded using a microplate spectrophotometer (Labsystems iEMS Reader MF). Optical density measurements were averaged and converted to mM nitrites using a standard curve of sodium nitrite.

#### 4.3.7. Arginase 1 Activity Assay

The enzymatic activity of arginase 1 was determined in macrophage cultures isolated from the spleen. In order to assess the ARG levels, standard kit (Cat. No. MAK112, Sigma-Aldrich, St. Louis, MO, USA) was used. Samples were centrifuged, then a supernatant (40 μL), as well as urea standard working solution (50 μL, 1 mM) were added to the wells of a 96-well plate. Then, 10 μL of 5 × substrate buffer (ARG buffer and Mn solution) was added into each well and incubated at 37 °C for 2 h. Afterwards, a prepared urea reagent (200 μL) was added to all wells to stop the enzymatic reaction (60 min at room temperature). An ELISA-reader was used to read the absorbance at 430 nm. The arginase activity was based on the ARG amount that converted L-arginine to 1.0 μmol of urea per min at 37 °C.

#### 4.3.8. Cytokine Th1, Th2 and Th17 Quantification

Serum supernatant was used for cytokine quantification by ELISA according to the manufacturer’s instructions (Multi-Analyte ELISArray kit, Qiagen, Hilden, Germany) for mouse. Standard curves for each cytokine, from each kit, were generated by using the reference cytokine concentrations supplied by the manufacturers. The cytokine concentration in the unknown samples was determined from the absorbance read on a Microplate Reader (Labsystems iEMS Reader MF) at 450 and 570 nm and by comparing the absorbance of the samples with the standard curve.

#### 4.3.9. Measurement of VEGF Levels by ELISA

We used ELISA assay to measure the variation of VEGF levels in serum supernatant of mice bearing CT26.WT tumors. The supernatant was collected from all treatment groups. The VEGF level serum supernatant were measured by ELISA kit (Mouse VEGF Immunoassay, Quantikine^®^ ELISA, Catalog Number MMV00, R&D Systems Europe, Ltd.) according to the manufacturer’s instructions. Briefly, ascites fluid and homogenated tumor cell samples were diluted 1:2 and added to the precoated wells. Unbound substrate was washed away, and an enzyme-linked polyclonal antibody specific for mouse VEGF was added to the wells followed by subsequent washes and addition of color substrate. Individual well absorbance was measured at 450 nm using Microplate Reader (Labsystems iEMS Reader MF) with the correction wavelength set at 540 nm. A total of three independent experiments, each in triplicates, were assayed, and the mean VEGF protein level from each triplicate was used for statistical analysis.

#### 4.3.10. Measurement of MMP-2 and MMP-9 by ELISA

An ELISA assay was used to determine changes in MMP-2 and MMP-9 levels in the serum supernatant. The level of MMP-2 and MMP-9 was determined using the ELISA kit Mouse MMP-2 ELISA Kit and Mouse MMP-9 ELISA Kit manufactured by Chongqing Biospes Co., Ltd., Biospes, (Chongqing, China) according to the manufacturer’s instructions. Samples were diluted with buffer. A mean concentration of target protein of 40–400 ng/mL was selected and obtained by dilution, adding 10 μL of samples and 90 μL of standard dilution buffer. 100 μL of standard or appropriate sample was added to a 96-well microtiter plate and incubated for 90 min at 37 °C to allow binding of MMP-2 or MMP-9 to the immobilized antibody. Unbound substrate was washed away, and an enzyme-linked polyclonal antibody specific for mouse MMP-2 or MMP-9 was added to the wells followed by subsequent washes and addition of color substrate. The absorbance values of the samples were read at 450 nm on a Microplate reader Model 550 from Bio-Rad, and the mean MMP-2 and MMP-9 protein levels from each triplicate was used for statistical analysis.

#### 4.3.11. Comet Assay

In the study, we used the standard performance of a comet assay under alkaline conditions on leukocytes and hepatocytes of treated animals [100]. Cell suspension was embedded in agarose matrix (Sigma, St. Louis, MO, USA), and lysed (2.5 M NaCl, 100 mM Na_2_EDTA, 10 mM Tris, 1% Na-sarcosinate (Sigma), 1% Triton X-100 (Sigma), 10% DMSO (Kemika, Zagreb, Croatia), pH 10). After the lysis the slides were placed into alkaline solution (300 mM NaOH, 1 mM Na_2_EDTA, pH 13) and subsequently electrophoresed. Finally, the slides were neutralized in 0.4 M Tris buffer (pH 7.5). After the last wash, the preparations were fixed in 96% ethanol for 7 min, and the slides were air dried. On the day of analysis, the preparations were stained with 100 μL of ethidium bromide (20 μg/mL) for 10–12 min in the dark. The stained gels were washed briefly in Tris-HCl buffer, pH = 7.5, and covered with a coverslip. Stained specimens were analyzed using a Leitz Wetzlar epifluorescence microscope and a COHU High Performance CCD Camera, MOD 4912-5000/0000 with a 515‒560 nm excitation filter showing comet-like structures originating from the contours of the cell nuclei in the gel. Measurements of tail length, % DNA in tail and tail moment were performed using the Comet Assay II image analysis program, manufactured by Perceptive Instruments Ltd. We analyzed 100 comets (*n* = 4) per each specimen, i.e., a total of 400 cells from each experimental group.

### 4.4. Statistical Analysis

The data was presented as mean ± standard deviation (SD) of the representative experiment from three independent experiments. All data were analysed by Kruskal–Wallis ANOVA. Further analysis of the differences between the groups was made with multiple comparisons of mean ranks for all groups. Statistical analyses were performed using STATISTICA 12 software (StatSoft, Tulsa, OK, USA). The data were considered significant at *p* < 0.05.

## Figures and Tables

**Table 1:** Composition of blended mushroom extracts.

Extract	Dry Matter Content (%)	Soluble Polysaccharides (mg/g of Dry Matter)	Total Polyphenols (mg GAE/L)	Total Flavonoids (mg GAE/L)
Agarikon Plus	5.05 ± 0.03	509.61	1457.8	750.9
Agarikon.1	93.00 ± 0.32	1053.88	448.75	240.83

**Table 2:** Induction of apoptosis in HCT-116 and SW620 cells after treatment with Agarikon Plus (AP), Agarikon.1 (AG.1) or their combination 1:1 (AP + AG.1) ^a^.

Cell Line	Duration of Treatment	Concentration	Early Apoptosis (%)	Late Apoptosis (%)	Necrosis (%)
HCT-116	24 h	Control	7.2 ± 0.7	2.46 ± 1.09	3.17 ± 1.01
		AP c_1_	16.24 ± 2.7	4.12 ± 1.01	2.34 ± 1.06
		AP c_2_	8.34 ± 1.88	3.3 ± 1.21	5.07 ± 2.42
		AP c_3_	5.7 ± 0.47	3.24 ± 1.81	3.36 ± 0.92
		AG.1 c_2_	44.3 ± 8.5 **	10.1 ± 0.51 **	0.82 ± 0.81
		AG.1 c_3_	21.01 ± 5.97	11.78 ± 2.55 **	2.44 ± 2.25
		AP + AG.1 c_1_	46.56 ± 14.8 **	4.5 ± 1.62	0.55 ± 0.53
		AP + AG.1 c_2_	23.6 ± 3.3	3.04 ± 0.66	2.04 ± 0.42
		AP + AG.1 c_3_	13.71 ± 6.03	5.8 ± 2.33	4.17 ± 1.45
SW620	24 h	Control	5.23 ± 1.6	2.22 ± 1.22	1.7 ± 1.43
		AP c_1_	14.57 ± 1.3	2.62 ± 0.63	0.81 ± 0.58
		AP c_2_	15.26 ± 1.83	3.53 ± 0.68	1.41 ± 0.6
		AP c_3_	5.69 ± 0.86	3.34 ± 0.53	1.97 ± 0.82
		AG.1 c_2_	50.31 ± 5.05 ***	4.48 ± 1.59	0.19 ± 0.28
		AG.1 c_3_	30.05 ± 4.32	4.83 ± 1.59	0.66 ± 0.94
		AP + AG.1 c_1_	45.98 ± 5.08 ***	6.94 ± 2.63 **	0.79 ± 0.98
		AP + AG.1 c_2_	31.07 ± 2.56	4.71 ± 0.96	0.49 ± 0.25
		AP + AG.1 c_3_	9.34 ± 0.92	3.74 ± 0.63	2.72 ± 0.2

^a^ Induction of apoptosis was analyzed after Annexin V and PI staining protocol for flow cytometry. The cells were sorted into intact cells (Annexin V−PI−), early apoptotic cells (Annexin V+ PI−), late apoptotic cells (Annexin V+PI+), and necrotic cells (Annexin V−PI+). Values are the means ± SD of three independent experiments. * Significantly different ** *p* < 0.01; *** *p* < 0.001) in comparison with control group. The following dilutions were applied: c_1_ (100×); c_2_ (1000×); c_3_ (10,000×). The values for AG.1 c_1_ could not be read due to precipitate formation. AP, Agarikon Plus; AG.1, Agarikon.1; 5-FU, 5-fluorouracil.

**Table 3:** Survival analysis of Balb/c mice bearing CT.26.WT after treatment.

Experimental Group Analysis	Overall Survival Rates	Kaplan–Meier	
**Curative Subsection**	**% of Survivors on Day 55 after Tumor Inoculation**	***p***	**χ^2^**
Control	0	NA	NA
AP	22.2	0.777	0.080
AP + 5-FU	87.5	0.000	11.206
AG.1	50	0.065	3.404
AG.1 + 5-FU	33.3	0.018	5.512
AP + AG.1	55.5	0.022	5.217
AP + AG.1 + 5-FU	50	0.001	10.288
5-FU	77.7	0.002	8.948
**Preventive Subsection**	**% of Survivors on Day 45 after Tumor Inoculation**	***p***	**χ^2^**
AP	100	NA	NA
AG.1	100	NA	NA

AP, Agarikon Plus; AG.1, Agarikon.1; 5-FU, 5-fluorouracil; NA, not applicable, survival of control group based on literature.

**Table 4:** Production of nitric oxide (NO) and arginase 1 activity in supernatant of spleen macrophages from mice bearing CT26.WT tumors.

Experimental Group ^a^	NO Levels (μM)	Arginase 1 Activity (U/L)
Control	1.62 ± 1.08	7.09 ± 1.26
AP	2.86 ± 0.64	3.78 ± 0.28
AP + 5-FU	15.55 ± 0.99 ***	2.97 ± 0.29 ***
AG.1	1.76 ± 0.54	3.59 ± 0.11 *
AG.1 + 5-FU	2.6 ± 0.54	5.01 ± 0.4
AP + AG	4.55 ± 0.5	3.73 ± 0.34
AP + AG + 5-FU	3.9 ± 0.3	3.71 ± 0.89
5-FU	9.9 ± 4.17 **	2.47 ± 1.04 ***

^a^ Mice were injected s.c. with CT26.WT (1 × 10^6^/mouse) cells and treated. Mice were sacrificed on day 28 post tumor cell inoculation and spleen macrophages were obtained as described in Materials and methods. Values are the means ± SD of three independent experiments. * Significantly different (* *p* < 0.05; ** *p* < 0.01; *** *p* < 0.001) in comparison with control group.

**Table 5:** Effect of treatment with Agarikon Plus, Agarikon.1 and 5-fluorouracil in various combinations on proangiogenic growth factor VEGF levels in serum supernatant of mice bearing CT26.WT tumors.

Experimental Group ^a^	VEGF (pg/mL)
Control	51.60 ± 6.39
AP	21.78 ± 6.88 *
AP + 5-FU	31.02 ± 0.82
AG.1	23.28 ± 10.15
AG.1 + 5-FU	26.14 ± 6.24
AP + AG	21.86 ± 4.44 **
AP + AG + 5-FU	14.67 ± 5.23 ***
5-FU	34.61 ± 7.87

^a^ Mice were injected s.c. with CT26.WT (1 × 10^6^/mouse) cells and treated. Mice were sacrificed on day 28 post tumor cell inoculation and VEGF levels in serum supernatant were measured by ELISA kit (Mouse VEGF Immunoassay, Quantikine^®^ ELISA, Catalog Number MMV00, R&D Systems Europe, Ltd., Abingdon, United Kingdom) according to the manufacturer’s instructions. Values are the means ± SD of three different observations. * Significantly different (* *p* < 0.05; ** *p* < 0.01; *** *p* < 0.001) in comparison with control group.

**Table 6:** Effect of treatment with Agarikon Plus, Agarikon.1 and 5-fluorouracil in various combinations on MMP-2 and MMP-9 levels in serum supernatant of mice bearing CT26.WT tumors.

Experimental Group	MMP-2 (ng/mL)	MMP-9 (ng/mL)
Control	245.01 ± 26.72	201.49 ± 33.06
AP	232.69 ± 7.29	284.76 ± 50.42
AP + 5-FU	264.38 ± 30.03	180.90 ± 43.38
AG.1	285.25 ± 38.71	260.02 ± 28.52
AG.1 + 5-FU	331.19 ± 20.81 **	155.16 ± 36.30
AP + AG	294.87 ± 10.03	206.62 ± 4.54
AP + AG + 5-FU	270.51 ± 20.13	179.86 ± 34.39
5-FU	292.66 ± 36.79	164.38 ± 44.93

Mice were injected s.c. with CT26.WT (1 × 10^6^/mouse) cells and treated. Mice were sacrificed on day 28 post tumor cell inoculation and MMP-2 and MMP-9 levels in serum supernatant were measured by ELISA kit (Chongqing Biospes Co., Ltd., Biospes, China) according to the manufacturer’s instructions. Values are the means ± SD. * Significantly different (** *p* < 0.01) in comparison with control group.

**Table 7:** Effect of treatment with Agarikon Plus, Agarikon.1 and 5-fluorouracil in various combinations on comet assay parameters as an indicator of DNA damage on peripheral leukocyte cells of Balb/c mice.

Peripheral Leukocyte Cells Comet Assay Parameters						
Group	Tail Length (μm)	FC	Tail Intensity	FC	Tail Moment	FC
Control	14.55 ± 0.24	0.00	2.88 ± 0.37	0.00	0.34 ± 0.04	0.00
AP	15.97 ± 0.30 ***	1.09	6.41 ± 0.72	2.22	0.73 ± 0.08	2.14
AP + 5-FU	16.59 ± 0.31 ***	1.14	5.27 ± 0.46 ***	1.82	0.65 ± 0.06 ***	1.91
AG	17.64 ± 0.25 ***	1.21	9.16 ± 0.73 ***	3.18	1.10 ± 0.08 ***	3.23
AG + 5-FU	14.33 ± 0.26	−0.02	2.64 ± 0.33	−0.08	0.31 ± 0.03	−0.08
AP + AG	16.05 ± 0.27 ***	1.10	5.49 ± 0.54	1.90	0.64 ± 0.06	1.88
AP + AG + 5-FU	16.98 ± 0.32 ***	1.16	3.49 ± 0.36	1.21	0.48 ± 0.04	1.41
5-FU	18.91 ± 0.36 ***	1.29	7.49 ± 0.73 ***	2.60	1.04 ± 0.10 ***	3.05

100 comets (*n* = 4) per each specimen were analyzed, i.e., a total of 400 cells from each experimental group. Data is expressed as mean ± SE. * Significantly different (*** *p* < 0.001) in comparison with control group. FC—fold change in comparison with control group.

**Table 8:** Effect of treatment with Agarikon Plus, Agarikon.1 and 5-fluorouracil in various combinations on comet assay parameters as an indicator of DNA damage on hepatocytes in Balb/c mice.

Hepatocyte Comet Assay Parameters						
Group	Tail Length (μm)	FC	Tail Intensity	FC	Tail Moment	FC
Control	14.35 ± 0.26	0.00	2.69 ± 0.33	0.00	0.32 ± 0.04	0.00
AP	15.32 ± 0.29	1.06	3.05 ± 0.31	1.13	0.38 ± 0.03	1.18
AP + 5-FU	17.88 ± 0.33 ***	1.24	4.73 ± 0.46 ***	1.75	0.67 ± 0.06 ***	2.09
AG	14.77 ± 0.29	1.02	3.78 ± 0.48	1.40	0.45 ± 0.05	1.40
AG + 5-FU	13.56 ± 0.26	−0.06	1.58 ± 0.32 ***	−0.41	0.18 ± 0.03 ***	−0.44
AP + AG	16.53 ± 0.28 ***	1.15	5.22 ± 0.51 ***	1.94	0.64 ± 0.06 **	2
AP + AG + 5-FU	16.75 ± 0.31 ***	1.16	4.55 ± 0.51	1.69	0.58 ± 0.06	1.81
5-FU	17.44 ± 0.38 ***	1.21	4.21 ± 0.52	1.56	0.59 ± 0.07	1.84

100 comets (*n* = 4) per each specimen were analyzed, i.e., a total of 400 cells from each experimental group. Data is expressed as mean ± SE. * Significantly different (** *p* < 0.01; *** *p* < 0.001) in comparison with control group. FC—fold change in comparison with control group.
